# The association between serum CD4 T lymphocyte counts and surgical outcomes in HIV/AIDS patients in Guangxi, China: a retrospective cohort study

**DOI:** 10.7717/peerj.12023

**Published:** 2021-09-20

**Authors:** Aimei Liu, Cunxu Liu, Xiaojun Deng, Yongbao Huang, Linchu Liao, Zhihao Meng, Minfu He, Junli Huang

**Affiliations:** 1 Longtan Hospital of Guangxi Zhuang Autonomous Region, Liuzhou, Guangxi, China; 2 Infectious Disease Medical Quality Control Center, Liuzhou, Guangxi, China; 3 Orthopedics, Longtan Hospital of Guangxi Zhuang Autonomous Region, Liuzhou, Guangxi, China; 4 General Surgery, Longtan Hospital of Guangxi Zhuang Autonomous Region, Liuzhou, Guangxi, China; 5 Infection, Longtan Hospital of Guangxi Zhuang Autonomous Region, Liuzhou, Guangxi, China

**Keywords:** Outcome, Surgical, HIV/AIDS, CD4T lymphocyte

## Abstract

**Background:**

HIV/AIDS is a chronic disease leading to complications in infected individuals that often require surgical intervention. These patients’ serum CD4 T lymphocyte (CD4) counts represent one of the most important indicators of their ability to tolerate surgical treatment. Previous studies have demonstrated that CD4 cell count (CD4-CC) < 200 cells/μl may increase the risk of surgical complications in these patients, limiting their ability to undergo surgery, which may negatively affect their quality of life. Further investigation into the surgical outcomes of patients with CD4-CC < 200 cells/μl should provide guidance in making appropriate clinical decisions for the optimal healthcare of this patient demographic.

**Methods:**

All enrolled patients were selected from 14 prefecture-level general hospitals in Guangxi, China, and were referred to AIDS outpost hospitals for inpatient surgical therapy. A total cohort of 168 adult patients was retrospectively analyzed. Multifactorial and stratified analyses were performed to evaluate the in surgical outcome differences for patients with CD4-CC < 200 cells/μl (*N* = 43), using those with CD4-CC ≥ 200 cells/μl (*N* = 125) as controls.

**Results:**

Poor incisional healing was used as the primary outcome indicator, and postoperative complications were used as the secondary outcome indicator. In the patient group with CD4-CC < 200 cells/μl, the risk of surgical complications was significantly increased (OR 2.379; 95% CI [1.049–5.394]) after adjustment. Adjusted stratified analysis of the CD4-CC < 200 cells/μl group revealed that individuals over 60 years (OR 27.504; 95% CI [2.297–329.317]) with erythrocyte counts below 4.00/ml for males or 3.50/ml for females (OR 3.353; 95% CI [1.079–10.419]) had a significantly higher risk of postoperative complications; this finding was statistically different from the control (CD4 ≥ 200 cells/μl) group. However, there was no significant difference between the two groups regarding the risk of poorly healed incision outcomes.

**Conclusions:**

Preliminary findings suggest that a serum CD4-CC < 200 cells/μl is not a definitive contraindication for surgical therapy and that baseline and surgical characteristics may help predict surgical outcomes in these patients. Further studies are needed to confirm these findings.

## Introduction

### Background

The implementation of highly active antiretroviral therapy (ART) in 1995 has resulted in a longer life expectancy in HIV/AIDS patients, and it is estimated that 20% to 25% of HIV/AIDS patients will require elective or emergency surgery at some point in time (Dua, Wajed & Winslet, 2007). According to the Chinese Center for Disease Control and Prevention (CDC) report, as of 2019, a total of 79,000 people living with the human immunodeficiency virus (PLWH) were under treatment in Guangxi, China. With a treatment coverage rate of 83.63%, up to 89.14% of these patients have been receiving therapy for more than 12 months (Public Health Sciences Data Center (China), 2019). Moreover, the overall mortality rate of patients under treatment was 2.63 per 100 persons a year (Chen et al., 2019). High treatment coverage rates and continued treatment rates have led to an increasing, significantly longer life expectancy in this population and an increase in the number of HIV/AIDS-related or non-related conditions requiring surgical treatment, including some immunocompromised populations that require surgical treatment. According to Motaner’s staging system (WHO, 1990), indications for major surgery on HIV-infected patients do not include the CD4-CC < 200 cells/μl group but principally focus on palliative purposes. In order to improve the quality of life of PLWH, over the last four years, hospitals specializing in the treatment of AIDS have been gradually implementing surgical interventions for PLWH who have not been under HAART (or for whom ART has failed) and in those with CD4-CC < 200 cells/μl. Therefore, this study used the group consisting of PLWH with CD4-CC ≥ 200 cells/μl as a reference, we retrospectively studied the postoperative complications plus incisional healing in the CD4-CC < 200 cells/μl group and evaluated the differences in surgical outcomes between the two groups. Stratified analysis of the CD4-CC < 200 cells/μl group allows for a more accurate assessment of factors influencing surgical outcomes, revealing insights into effective ways of providing more personalized, comprehensive medical interventions for these patients.

### Objectives

Our primary objective was to compare the differences between patients with CD4-CC ≥ 200 cells/μl and those with CD4-CC < 200 cells/μl in terms of demographic data, preoperative baseline, surgical characteristics, and surgical outcomes for each variable. We also aimed at evaluating the impact of stratification factors on surgical outcomes such as postoperative complications and incisional healing. Data on the retrospective surgical baseline and surgical characteristics were obtained from the hospital HIS system.

## Methods

### Ethics statement

The study and use of systematic data were approved by the Ethics committee of Longtan Hospital of Guangxi Zhuang Autonomous Region and the AIDS Medical Quality Control Center in Guangxi. Written informed consent was obtained from all of the participants after a full explanation of the study.

### Study design

Based on the retrospective analysis of data from the hospital Hospital Information System (HIS), patients were divided into two groups based on their preoperative CD4-CC:CD4-CC < 200 cells/μl group (the observation group) and CD4-CC ≥ 200 cells/μl group (the control group). A multifactorial analysis was conducted to compare the demographic data, preoperative baseline, surgical characteristics, and surgical outcomes in both groups in order to assess the differences in surgical complication risks and poor incisional healing. The multifactorial analysis also assessed the effects of stratification factors on surgical outcomes.

### Research objectives

1. On the day of attendance, venous blood hemocyto analysis was measured at the clinical group of the Clinical Laboratory Department of Longtan Hospital of Guangxi Zhuang Autonomous Region, used a fully active hematology analyzer (XN-1000, SYSMEX) to detect erythrocyte and hemoglobin. Two ml of venous blood was extracted, which was added into premixed anticoagulant (EDTA-K2) of the vacuum sampling vessel and mixed evenly. The instrument was opened for automatic operation. Erythrocyte and hemoglobin assays were measured using daily internal quality control procedures to assess imprecision and monthly external quality control procedures to assess accuracy.
2. In the morning of the second day of hospitalization, plasma albumin was performed on an empty stomach, and was measured at the clinical biochemistry group of Longtan Hospital of Guangxi Zhuang Autonomous Region. Three ml of venous blood was extracted and added into a vacuum sample tube with a premixed anticoagulant (Heparin lithium salt), and mixed evenly, centrifuged at low speed (3,000 rpm/min), and the automatic biochemical apparatus (Abbott Laboratories c16000) was opened and operated automatically. Plasma albumin was measured using daily internal quality control procedures to assess imprecision and monthly external quality control procedures to assess accuracy.
3. On the day of attendance, Venous blood flow cytometry (FCM) was measured at the Department of Clinical Immunology in Longtan Hospital of Guangxi Zhuang Autonomous Region, used Flow Cytometer (CanntoII, BD). A total of 20 μl LCD3/CD8/CD45/CD4 antibody reagent and 50 μl EDTA-K2 fully mixed whole blood was added to the TuCount tube, which was mixed and left for 15–20 min at room temperature and away from light. The tube was taken out and 450 μl diluted FACS hemolysin was added, mixed well, and placed away from light for 10 min, then checked on the machine. FCM were measured using daily internal quality control procedures to assess imprecision and monthly external quality control procedures to assess accuracy.

### Entry criteria

1. Minimum age of 18 years.
2. PLWH with specific surgical indications between 2016 and 2019 were continuously included.
3. Disease diagnosis was made using the International Classification of Diseases, 10th edition of Clinical diagnosis and procedure codes (ICD-10). Including general surgery: A18.207, C16.900, D36.705, E05.900 × 001, I84.201, K31.814, K35.900, K40.900 × 002, K40.900 × 003, K61.001, K65.002, K80.501, M89.900 × 072, R10.000, etc; Bone and Joint Surgery: A18.007 + M49.0*, A18.034 + M01.1*, M51.204, M86.913, M87.800 × 051, M89.900 × 072, R02. × 00, S42.000. S42.301, S72.000, S72.101, S72.900, S82.000, S82.201, S82.202, S92.000, Z47.000 × 002, etc; Urology surgery: C60.900, K20.100, N40. × 00, N43.301, etc; Thoracic Surgery: A16.201, C50.900 × 011, etc.
4. According to ICD-9-CM, surgery codes are as follows: General Surgery: 06.3900 × 004, 40.1103, 40.5100, 43.8901, 43.9901, 44.6901, 47.0901. 48.6903, 49.0100, 49.4500 × 002, 51.2200, 53.0401, 53.0501, 54.1100, 54.1900 × 010, 64.2 × 01, 66.6104, 83.3200 × 001, etc; Bone and Joint Surgery: 41.3800 × 001, 77.3907, 77.6903, 78.5600. 79.3101, 79.3500 × 016, 79.3501, 79.3600 × 017, 79.3601, 79.3603, 79.3700 × 013, 79.3900 × 001, 79.3900 × 051, 79.3904, 79.8903, 80.8601, 81.0500 × 006, 81.0502, 81.0800 × 016, 81.2200, 81.5100, 81.5200, etc; Urology Surgery: 55.0401, 55.1101, 56.0 × 04, 56.2 × 01, 57.0 × 00 × 013, 59.8 × 03, 59.9901, 60.2901, 64.3 × 01, etc; Thoracic Surgery: 32.2904, 34.5902, 85.4100 × 001.
5. Diagnosis of postoperative complications was based on the ICD-10. Patients were observed for two weeks depending on the surgical procedure to determine if they had developed any complications. Includes K82.300, J98.414, R19.001, I50.000, K63.212, N39.000, R33. × 00, T81.406, T81.811, K63.210, K92.210, R31. × 00, N50.800 × 023, K56.500 × 003, J94.801.

### Exclusion criteria

Patients with a history of organ transplantation or chronic immunosuppressive therapy were excluded if the drug administered interfered with the patient’s baseline test values.

Patients with incomplete data regarding demographics, surgical baseline, and surgical status were also excluded.

### Variables

1. Diagnosed with HIV/AIDS based on the diagnostic criteria of the Chinese CDC (National Health Commission of the people’s Republic of China, 2019).
2. The research variables and adjusted multivariate regression analysis were as follows:

Demographic data: Gender, age, educational background, occupation, ethnicity, route of infection, body mass index (BMI), etc. Patients with incomplete information on demographics, surgical baseline and surgical status were excluded.

Surgical baseline: Erythrocyte counts, hemoglobin levels, plasma albumin, ART treatment time, comorbidities, flow cytometry, etc.

Surgical characteristics: Type of surgeries involved, including emergency surgeries or elective surgeries. Disease types listed in the inclusion criteria covering general surgery, orthopedic joint surgery, urology, thoracic surgery. Surgical classification can be divided into one to four classes of surgery based on the degree of difficulty and risk of each type of surgery, incision classification including clean, potentially contaminated, and contaminated incisions (Class I to Class III incisions). The types of surgery consisted of minimally invasive surgery and conventional surgery.

Baseline surgical complications, surgical classification, incisional classification and postoperative complications were defined by criteria provided by the National Health and Wellness Commission of China.

### Outcome indicators

HIV/AIDS patients with CD4-CC ≥ 200 cells/μl who underwent surgery were used as a control group and compared to those with CD4-CC < 200 cells/μl. Poor incisional healing was used as the primary outcome indicator, and postoperative complications were used as the secondary outcome indicator. In contrast, multifactorial regression analysis was performed to assess risk differences in primary and secondary outcome indicators for the overall and stratified patients.

### Statistical analysis

All of the statistical analyses were performed using the Statistical Analysis System (SPSS), version 26.0. Categorical variables were studied using a single-factor analysis. Independent variables with *P*-values <0.05 for the single-factor analysis were included in the multivariate analysis. Subsequently, a multiple regression analysis was used to compare the overall and stratified independent variables with the outcome indicators. The odds ratio (OR) was estimated with a 95% confidence interval (CI). Statistical significance was reached when *P* < 0.05.

## Results

A total of 168 hospitalized adult HIV/AIDS patients who met our inclusion criteria and underwent surgery from 2016 to 2019 were included in this study. The method of inclusion in this study is continuous inclusion, which solves the selective bias. Three postoperative outcomes occurred in these patients: Postoperative complications occurred in 66 cases (39.29%). Poor healing was reported for Grade B or C incisions in 17 cases (10.12%). Moreover, two deaths related to surgical disease or surgical complications were recorded within 2 weeks after surgery (1.19%). The number of deaths did not meet the statistical requirements and were not included in this study’s outcome measures.

Table 1 illustrates patient demographics and surgical baseline data. The majority of patients in this cohort were males (76.8%), mainly young adults (72.0%) ranging from 18 to 60 years old, and farmers (66.7%) with at least a basic educational (74.4%) background. The HIV/AIDS infection route for the vast majority of patients was sexual transmission (94.6%) in Guangxi, China, a multi-ethnic region, with ethnic minorities (35.1%) making up roughly one-third of the patients. This is consistent with the demographic data of AIDS patients in China (National Health Commission of the people’s Republic of China, 2019). The number of patients with erythrocyte counts of less than 4.00/ml or 3.50/ml in men or women respectively at baseline was 54.8%, and the number of patients with a hemoglobin level of less than 120 g/l and 110 g/l in men and women, respectively, was 41.7%. Meanwhile, approximately 26.2% of these patients had preoperative comorbidities. Furthermore, 18.5% of patients were treated with ART preoperatively for less than 30 days, a statistically different finding between the two groups (*P* = 0.021). Other preoperative baseline values such as gender (*P* = 0.037), occupation (*P* = 0.002), preoperative hemoglobin level (*P* = 0.029), and plasma albumin level (*P* = 0.049) also elicited statistically significant differences between the two groups. In terms of the remaining demographic information and baseline values such as age, educational background, ethnicity, route of infection, BMI, erythrocyte counts, preoperative comorbidities and CD4/CD8 variables, none of the differences were statistically significant between the two groups (*P* ≥ 0.05).

**Table 1:** Preoperative demographics and baseline of patients with different CD4 levels.

Item	Total (%) (*N* = 168)	CD4 ≥ 200 (*N* = 125)	CD4 < 200 (*N* = 43)	*P* value
Age				
18–60	121 (72.0)	91 (72.8)	30 (69.8)	0.702
>60	47 (28.0)	34 (27.2)	13 (30.2)	
Gender				
Male	129 (76.8)	91 (72.8)	38 (88.4)	0.037
Female	39 (23.2)	34 (27.2)	5 (11.6)	
Educational background				
Elementary and junior high school	125 (74.4)	90 (72.0)	35 (81.4)	0.223
High school and above	43 (25.6)	35 (28.0)	8 (18.6)	
Career				
Farmers	112 (66.7)	75 (60.0)	37 (86.0)	0.002
Other*	56 (33.3)	50 (40.0)	6 (14.0)	
Ethnicity				
Han	109 (64.9)	83 (66.4)	26 (60.5)	0.482
Zhuang and others**	59 (35.1)	42 (33.6)	17 (39.5)	
Route of infection				
Sexual transmission	159 (94.6)	118 (94.4)	41 (95.3)	1.000
Intravenous drug injection	9 (5.4)	7 (5.6)	2 (4.7)	
Body mass index				
<18.5	37 (22.0)	29 (23.2)	8 (18.6)	0.409
18.5 ≤ BMI* ≤ 24	112 (66.7)	80 (64.0)	32 (74.4)	
>24	19 (11.3)	16 (12.8)	3 (7.0)	
Erythrocyte				
Female ≥ 4.00/ml, or male ≥ 3.50/ml	76 (45.2)	57 (45.6)	19 (44.2)	0.872
Female < 4.00/ml, or male < 3.50/ml	92 (54.8)	68 (54.4)	24 (55.8)	
Hemoglobin				
Female ≥ 120, or male ≥ 110 g/L	98 (58.3)	79 (63.2)	19 (44.2)	0.029
Female < 120, or male < 110 g/L	70 (41.7)	46 (36.8)	24 (55.8)	
Plasma albumin				
<35	141 (83.9)	109 (87.2)	32 (74.4)	0.049
≥35	27 (16.1)	16 (12.8)	11 (25.6)	
Treatment duration of HARRT				
<30 days	31 (18.5)	18 (14.4)	13 (30.2)	0.021
≥30 days	137 (81.5)	107 (85.6)	30 (69.8)	
Complication				
Without	124 (73.8)	94 (75.2)	30 (69.8)	0.485
With	44 (26.2)	31 (24.8)	13 (30.2)	
CD4/CD8				
≤1.0	154 (91.7)	112 (89.6)	42 (97.7)	0.098
>1.0	14 (8.3)	13 (10.4)	1 (2.3)	

**Notes:**

* Other occupation refers to: 16 cases of freelance, 12 cases of unemployment, 11 cases of retirement, six cases of self-employed, five cases of workers, three cases of staff, two cases of teachers and students, one case of detainees.

** Other ethnicity refers to: 54 in Zhuang, three in Miao, one in Dong and one in Mulao.

Table 2 delineates the comparison of surgical characteristics between the two groups. With regard to the disease-specific subgroup analysis, orthopedic and joint surgery was more likely to be treated surgically (43.5%). Grade-III surgery with more complex procedures, higher technical requirements, and higher risks had the highest proportion in both groups (34.9% in the CD4-CC < 200 cells/μl group, and 46.4% in the CD4-CC ≥ 200 cells/μl group). It is also worth noting that these HIV/AIDS patients were more likely to undergo traditional surgical approaches (80.95%). The proportion of contaminated incisions in the CD4-CC < 200 group was significantly higher than that in the CD4-CC > 200 group and proved to be significantly different (*P* = 0.001). Other variables of surgical characteristics, such as the type of admission, type of disease, surgical grade, and minimally invasive surgery, did not display any statistically significant difference between the two groups.

**Table 2:** Comparison of surgical characteristics of HIV/AIDS patients with different CD4.

Item	Total (*N* = 168)	CD4 ≥ 200 (*N* = 125)	CD4 < 200 (*N* = 43)	*P* value
Type of surgery of admission				
Emergency surgery	78 (46.4)	57 (45.6)	21 (48.8)	0.714
Elective surgery	90 (53.6)	68 (54.4)	22 (51.2)	
Type of disease*				
Forensic sursery	62 (36.9)	44 (35.2)	18 (41.9)	0.393
Orthopaedic surgery	73 (43.5)	58 (46.4)	15 (34.9)	
Urology	30 (17.9)	20 (16.0)	10 (23.3)	
Thoracic surgery	3 (1.7)	3 (2.4)	0	
Surgical grading**				
Grade I surgery	16 (9.5)	11 (8.8)	5 (11.6)	0.803
Grade II surgery	59 (35.1)	45 (36.0)	14 (32.6)	
Grade III surgery	68 (40.5)	49 (39.2)	19 (44.2)	
Grade IV surgery	25 (14.9)	20 (16.0)	5 (11.6)	
Classification of incision				
clean incision	101 (60.1)	86 (68.8)	15 (34.9)	0.001
Possible contaminated and contaminated incisions	67 (39.9)	39 (31.2)	28 (65.1)	
Minimally invasive surgery				
Yes	32 (19.05)	22 (17.6)	10 (23.3)	0.415
No	136 (80.95)	103 (82.4)	33 (76.7)	

**Notes:**

* General Surgery *vs*. Orthopaedic Surgery = 0.253, General Surgery *vs*. Urology = 0.674, General Surgery *vs*. Thoracic Surgery = 0.555.

** Grade I surgery *vs*. Grade II surgery = 0.772, Grade I surgery *vs*. Grade III surgery = 1.000 and Grade I surgery *vs*. Grade IV surgery = 0.656.

Table 3 lists the risk-adjusted surgical outcomes. After adjusting for surgical baseline data such as gender, age, body mass index, erythrocyte counts, duration of ART, comorbidities and flow cytometry, as well as surgical characteristics including the type of surgery, type of disease, surgical procedure, surgical grade and incision classification, we found that the patients in the CD4-CC < 200 cells/μl group were 2.379 times more likely to experience postoperative complications (*P* = 0.038). The risk-adjusted *P*-value for poor incisional healing was lower in the CD4-CC < 200 cells/μl group, but the difference did not reach a statistical significance.

**Table 3:** Analysis of surgical outcomes.

	Unadjusted			Adjusted		
	OR	95% CI	*P* value	OR	95% CI	*P* value
Complication						
≥200	2.496	[1.231–5.063]	0.010	2.379	[1.049–5.394]	0.038
<200						
Poor healing of the incision						
≥200	1.239	[0.410–3.745]	0.930	0.585	[0.162–2.113]	0.413
<200						

Table 4 demonstrates the single factor analysis stratification of the two groups. In the age stratification group, we found out that patients in the CD4-CC < 200 cells/μl group were 4.125-fold more likely to have postoperative complications than those in the CD4-CC ≥ 200 cells/μl group for patients older than 60 years old (*P* = 0.036). Erythrocyte counts stratification results revealed a 4.476-fold increase in the odds of postoperative complications ofthe CD4-CC < 200 cells/μl group in the subgroup below 4.00/ml in men or 3.50/ml in women (*P* = 0.002). Regarding emergency admissions, the odds of postoperative complications were 3.700 times higher in the CD4-CC < 200 cells/μl group than the CD4-CC ≥ 200 cells/μl group (*P* = 0.013). Stratified comparisons of hemoglobin levels and incisional classification were not statistically significant for the occurrence of postoperative complications. Similarly, stratified comparisons of age, erythrocyte counts, hemoglobin levels, incisional classification, and the type of admission were not statistically significant for incisional healing.

**Table 4:** Surgical complications and poor incision healing stratified by univariate analysis.

Stratification	Number of people	Number of people with complications (%)	OR (95% CI)	*P*	Number of people with poor incision healing (%)	OR (95% CI)	*P* value
Stratification 1: Age = “18–60”							
≥200	91	30 (33.0%)	1	0.094	9(9.9%)	1	*P* = 1.000
<200	30	15 (50.0%)	2.033 [0.879–4.703]		3 (10.0%)	1.012 [0.255–4.012]	
Stratification 1: Age = “>60”							
≥200	34	12 (35.3%)	1	0.036	3 (8.8%)	1	*P* = 0.902
<200	13	9 (69.2%)	4.125 [1.046–16.263]		2 (15.4%)	1.879 [0.276–12.775]	
Stratification 2: Erythrocytes = male ≥ 4.00/ml, or female ≥ 3.50/ml							
≥200	57	21 (36.8%)	1	*P* = 0.683	4 (7.0%)	1	*P* = 0.195
<200	19	8 (42.1%)	1.247 [0.433–3.591]		4 (21.1%)	3.533 [0.789–15.832]	
Stratification 2: Erythrocytes = male < 4.00/ml, or female < 3.50/ml							
≥200	68	21 (30.9%)	1	*P* = 0.002	8 (11.8%)	1	*P* = 0.498
<200	24	16 (66.7%)	4.476 [1.659–12.076]		1 (4.2%)	0.326 [0.039–2.754]	
Stratification 3: Hemoglobin = male ≥ 120 g/L, or female ≥ 110 g/L							
≥200	79	25 (31.6%)	1	*P* = 0.087	6 (7.6%)	1	*P* = 1.000
<200	19	10 (52.6%)	2.400 [0.867–6.641]		2 (10.5%)	1.431 [0.265–7.719]	
Stratification 3: Hemoglobin = male < 120 g/L, or female < 110 g/L							
≥200	46	17 (37.0%)	1	*P* = 0.087	6 (13.0%)	1	*P* = 1.000
<200	24	14 (58.3%)	2.388 [0.871–6.547]		3 (12.5%)	0.952 [0.216–4.197]	
Stratification 4: Incision classification = clean incision							
≥200	86	24 (27.9%)	1	*P* = 0.051	5 (5.8%)	1	*P* = 1.000
<200	15	8 (53.3%)	2.952 [0.965–9.035]		1 (6.7%)	1.157 [0.126–10.662]	
Stratification 4: Incision classification = possible contaminated or contaminated incision							
≥200	39	18 (46.2%)	1	*P* = 0.522	7 (17.9%)	1	*P* = 0.750
<200	28	16 (57.1%)	1.556 [0.585–4.137]		4 (14.3%)	0.762 [0.200–2.903]	
Stratification 5: Type of surgery of admission = elective							
≥200	68	22 (32.4%)	1	*P* = 0.264	8 (11.8%)	1	P=1.000
<200	22	10 (45.5%)	1.742 [0.653–4.647]		3 (13.6%)	1.184 [0.285–4.917]	
Stratification 5: Type of surgery of admission = emergency							
≥200	57	20 (35.1%)	1	*P* = 0.013	4 (7.0%)	1	*P* = 1.000
<200	21	14 (66.7%)	3.700 [1.285–10.657]		2 (9.5%)	1.395 [0.236–8.241]	

Table 5 presents a multifactorial analysis of patient stratification in both groups. After risk adjustment, the risk of complications in the subgroup older than 60 years with CD4-CC < 200 cells/μl was significantly increased compared with the univariate analysis (OR 27.504), which was statistically significant (*P* = 0.009). Additionally, the adjusted erythrocyte-count stratification indicated that the incidence of postoperative complications was still higher (OR 3.353) in the CD4-CC < 200 cells/μl group (*P* = 0.036) for both males and females. There was no statistically significant difference between emergency and elective surgeries after adjustment for stratified admission types.

**Table 5:** Surgical complications stratified by multifactorial analysis.

Stratification	Number of people	Number of people with complications (%)	OR (95% CI)	*P* value
Stratification 1: Age = “18–60”				
≥200	91	30 (33.0%)	1	0.343
<200	30	15 (50.0%)	1.587 [0.610–4.124]	
Stratification 1: Age = “>60”				
≥200	34	12 (35.3%)	1	0.009
<200	13	9 (69.2%)	27.504 [2.297–329.317]	
Stratification 2: Erythrocytes = male ≥ 4.00/ml, or female ≥ 3.50/ml				
≥200	57	21 (36.8%)	1	0.785
<200	19	8 (42.1%)	1.198 [0.327–4.383]	
Stratification 2: Erythrocytes = male < 4.00/ml, or female < 3.50/ml				
≥200	68	21 (30.9%)	1	0.036
<200	24	16 (66.7%)	3.353 [1.079–10.419]	
Stratification 3: Type of surgery of admission = elective				
≥200	68	22 (32.4%)	1	0.308
<200	22	10 (45.5%)	1.874 [0.560–6.274]	
Stratification 5: Type of surgery of admission = emergency				
≥200	57	20 (35.1%)	1	0.116
<200	21	14 (66.7%)	2.726 [0.782–9.503]	

## Discussion

HIV/AIDS is now widely recognized as a chronic disease rather than an acute and life-threatening illness. In Guangxi, China, late detection of HIV/AIDS >50.00% (Ge et al., 2019) and treatment interruptions account for 4.8 per 100 persons a year of the infected cases (Liu et al., 2018), and CD4 counts are becoming increasingly necessary for the evaluation of surgical outcomes. In this study, three characteristics of the CD4-CC < 200 cells/μl group were highlighted and compared to those of the control (CD4-CC ≥ 200 cells/μl) group. After a thorough analysis of the collected results, gender and occupation displayed a significant difference between the two groups of patients. This difference may indicate that male and rural patients with CD4-CC < 200 cells/μl have a lower willingness to seek medical care, leading to late detection of HIV/AIDS or decreased adherence to ART treatment. Early detection and early treatment of HIV/AIDS has been mandated as critical by the Chinese National Health authorities. Furthermore, clinical attention should be paid to enhance knowledge of the disease in these populations to improve treatment compliance. Next, patients in the two cohorts underwent surgery with different indications. Compared to patients in the CD4-CC ≥ 200 cells/μl group, those in the CD4-CC < 200 cells/μl group differed from the baseline in terms of less than 30 days of HARRT, low erythrocyte counts, and low plasma albumin. Infectious diseases account for about two-thirds, whereas the surgical characteristics were shown in 65.1% of the CD4 < 200 cells/μl group regarding the proportion of contaminated or potentially contaminated incisions. This discrepancy points out a worse preoperative composite in patients with CD4-CC < 200 cells/μl. Additionally, studies on abdominal surgery and plastic surgery have documented that CD4-CC < 200 cells/μl may negatively affect postoperative outcomes (Deneve et al., 2010; Davison et al., 2008). To this end, the surgical outcome of this study was designed in two distinct phases: Postoperative complications, which reflect the perioperative period, and incisional healing, which reflects the final surgical outcome. The results revealed that adjusted postoperative complications were statistically different between the two groups; however, no statistically significant difference was identified with respect to poor incisional healing. In other words, there was a statistically significant difference in the perioperative period after surgery in the CD4-CC < 200 cells/μl group, but no difference in endpoint outcomes. Also, our study further analyzed stratification factors with subgroups such as the elderly and erythrocytopenic patients, which had a significantly increased risk of postoperative complications in the CD4-CC < 200 cells/μl group. Previous studies have not reported stratification factors. Our study reports that CD4-CC is not a definitive contraindication for surgery; however, the patient’s baseline and surgical characteristics may help predict surgical outcomes and allow for appropriate interventions and intensive care. This study was conducted among HIV/AIDS patients over the age of 18, so it is not suitable for teenagers and children on the generalizability of the results.

Previous studies have found that the rate of postoperative infection in orthopedic joint surgeries among HIV-positive patients was 23%, and CD4-CC < 300 cells/μl, hospitalization, multiple traumas, and low plasma albumin levels were all found to be associated with postoperative infections (Guild et al., 2012). In our study, osteoarticular surgery was indicated for up to 43.5% of HIV/AIDS patients with 17 infectious and noninfectious diseases, including ICD-10 codes A18.007 + M49.0*, M87.800 × 051, and S72.000; these codes correspond to Pot’s disease (tuberculosis spondylitis), ischemic necrosis of the femoral head, and femoral fracture, respectively. Epidemiological data from Guangxi indicated that the proportion of HIV/AIDS patients with concurrent extrapulmonary tuberculosis was 8.86% (Huang et al., 2020); meanwhile, AIDS unrelated diseases and fractures also accounted for a large proportion of these orthopedic surgery cases. As a result, the proportion of osteoarticular surgeries is higher in our data.

Tuberculosis (TB) is a disease closely linked to cellular immunity, and it remains a common opportunistic infection in HIV/AIDS patients in Guangxi, China. Despite the fact that the incidence of tuberculosis has dropped from 120/100,000 in 2016 to 90/100,000, it is still a common opportunistic infection in HIV/AIDS patients (From 11.5% to 32.5%) (Pang et al., 2018; Xiao et al., 2013). In this study, 34 patients were operated on for infectious diseases, of which 15 TB and extrapulmonary TB-related surgeries were performed, accounting for 44.12% of these surgeries. ICD-10 codes A16.201, A18.007 + M49.0*, A18.207, A18.205, A18.034 + M01.1*, A18.118 + N51.1* and A18.300 × 009 were included on the list of diseases. This may be indicative of a higher prevalence of *Mycobacterium tuberculosis* infection among HIV/AIDS patients. Consequently, the medical management of this population must include regular screening and preventive measures for latent infections. Tuberculosis screening in China has been included in the annual screening program for HIV/AIDS patients by the Chinese National Health Administration. As far as we know, the AIDS outpost hospital where these surgeries were performed was the only provincial-level outpost hospital in the region, and its capacity was far superior to that of the prefecture-level outpost hospital. Hence, the more complex and risky Class III surgeries were referred from lower-level hospitals, which led to an increase in the incidence of Class III surgeries in this population. The incidence of Class III surgeries had indeed increased, mostly due to referrals from lower-level hospitals rather than an inherent rise of Class III surgeries in these people.

This study has some limitations, mainly due to flaws in its retrospective design. Chinese patients with HIV/AIDS can receive a free annual viral load test provided by the Chinese government. In this study, 46.4% of patients underwent emergency surgeries, and even some of those who had elective surgeries might also have had viral load testing performed more than 3 months prior to surgery, making it less relevant to surgical outcomes. Therefore, although it would have been interesting to examine the impact of viral load on surgical outcomes, we chose to rely on CD4-CC to determine the participants’ HIV/AIDS status. Additionally, the HIS system is unable to track patients who are automatically discharged within 2 weeks after surgery, and a small number of cases with missing data may often be cases with a poor prognosis in which the patient or family members chose to abandon treatment. Therefore, these cases were not included in this study, which may affect the accuracy of the data.

## Conclusions

The risk of surgical complications was significantly different in the CD4-CC < 200 cells/μl group compared to the CD4-CC ≥ 200 cells/μl group. Among the adjusted stratification factors, the risk of surgical complications was significantly increased in the elderly and erythrocytopenic patients in the CD4-CC < 200 cells/μl group, whereas no statistically significant difference was observed with respect to the risk of poorly healed incisions. A convincing explanation for this observation is that AIDS outpost hospitals have good diagnostic capabilities, and postoperative complications can be cured with medical intervention without affecting the final surgical outcome. This implies that CD4-CC are not a determinant factor in deciding to perform a surgical procedure but that the patients’ baseline and surgical characteristics may contribute to their surgical outcomes. Thus, appropriate interventions and intensive care should be administered when necessary. Notwithstanding, further studies are imperative to confirm these findings.

## Supplemental Information

10.7717/peerj.12023/supp-1 Supplemental Information 1 Source Material of Project: Systematic, sequential treatment of Comorbid Surgical Diseases in patients with HIV/AIDS.

## Additional Information and Declarations

### Data Availability

The following information was supplied regarding data availability: Raw data are available as a Supplemental File.
